# A Comparative Interaction between Copper Ions with Alzheimer's *β* Amyloid Peptide and Human Serum Albumin

**DOI:** 10.1155/2012/208641

**Published:** 2012-07-09

**Authors:** G. Rezaei Behbehani, L. Barzegar, M. Mohebbian, A. A. Saboury

**Affiliations:** ^1^Chemistry Department, Imam Khomeini International University, Qazvin 34149-16818, Iran; ^2^Chemistry Department, Faculty of Science, Islamic Azad University, Takestan Branch, Takestan 34819-49479, Iran; ^3^Institute of Biochemistry and Biophysics, University of Tehran, Tehran 14174, Iran

## Abstract

The interaction of Cu^2+^ with the first 16 residues of the Alzheimer's amyliod *β* peptide, *A*β** (1–16), and human serum albumin (HSA) were studied in vitro by isothermal titration calorimetry at pH 7.2 and 310 K in aqueous solution. The solvation parameters recovered from the extended solvation model indicate that HSA is involved in the transport of copper ion. Complexes between *A*β** (1–16) and copper ions have been proposed to be an aberrant interaction in the development of Alzheimer's disease, where Cu^2+^ is involved in *A*β** (1–16) aggregation. The indexes of stability indicate that HSA removed Cu^2+^ from *A*β** (1–16), rapidly, decreased Cu-induced aggregation of *A*β** (1–16), and reduced the toxicity of *A*β** (1–16) + Cu^2+^ significantly.

## 1. Introduction

One of the hallmarks of Alzheimer's disease is the accumulation of amyloid plaques between nerve cells (neurons) in the brain. Beta amyloid is a protein fragment snipped from an amyloid precursor protein (APP). In a healthy brain, these protein fragments are broken down and eliminated. In Alzheimer's disease, the fragments accumulate to form hard, insoluble plaques. Alzheimer's disease and heart disease were made worse by excess copper and iron. Researchers are still trying to fully understand how its plaques and tangles lead to memory loss and other symptoms and how to reverse those changes to prevent or stop the disease. However, there are treatments available today that can help patients manage the symptoms of Alzheimer's disease and delay its progression. Acetylcholine helps pass messages between certain brain cells involved in memory. In Alzheimer's disease, these brain cells start to die and the amount of acetylcholine is reduced. Cholinesterase inhibitors reduce the breakdown of acetylcholine and increase its levels in the brain. This reduces some of the symptoms of Alzheimer's disease. HSA carries metal ions, including physiological Ca^2+^, Zn^2+^, Co^2+^, and Cu^2+^, as well as toxic Cd^2+^ and Ni^2+^ [1–5]. 

Although the etiology of cognitive impairment in Alzheimer's disease (AD) is not fully understood, it has been reported that acetylcholine-producing neurons degenerate in the brains of patients with Alzheimer's disease. The degree of this cholinergic loss has been correlated with degree of cognitive impairment and density of amyloid plaque.

Galantamine, a tertiary alkaloid, is a competitive and reversible inhibitor of acetylcholinesterase. It is possible to postulate galantamine's action therapeutic effect by enhancing cholinergic function. This is accomplished by increasing the concentration of acetylcholine through reversible inhibition of its hydrolysis by cholinesterase. If this mechanism is correct, galantamine's effect may lessen as the disease process advances and fewer cholinergic neurons remain functionally intact. In humans, Cu^2+^ is necessary for the development of connective tissue, nerve coverings, and bone. Cu^2+^ also participates in both Fe and energy metabolism. Cu^2+^ acts as a reductant in the enzymes, superoxide dismutase, cytochrome oxidase, lysyl oxidase, dopamine hydroxylase, and several other oxidases that reduce molecular oxygen. Cu^2+^ deficiency in humans is rare, but when it occurs it leads to normocytic, hypochromic anemia, leucopenia and neuropenia, and inclusive osteoporosis in children. Excessive dietary Zn^2+^ can cause Cu^2+^ deficiency. Chronic Cu^2+^ toxicity is rare in humans, and mostly associated with liver damage. Curcumin has been implicated in resolving and preventing Alzheimer's disease-associated plaques or deposits both in vivo and vitro. Epidemiological data suggest that the consumption of curcumin is linked to a lower incidence of Alzheimer's disease. However, the solubility of curcumin in aqueous solutions is exceedingly low, which limits its systemic absorption and therapeutic potential. A new method is proposed to treat or prevent amyloid plaque formation associated with Alzheimer's disease by injection of highly concentrated blood serum-solubilized curcumin [2, 6–10]. The objective of this study was to assess the conformational changes of HSA and *Aβ*(1–16) due to their *Aβ*(1–16) binding to Cu^2+^ ion.

## 2. Materials and Method

**Figure 1:**
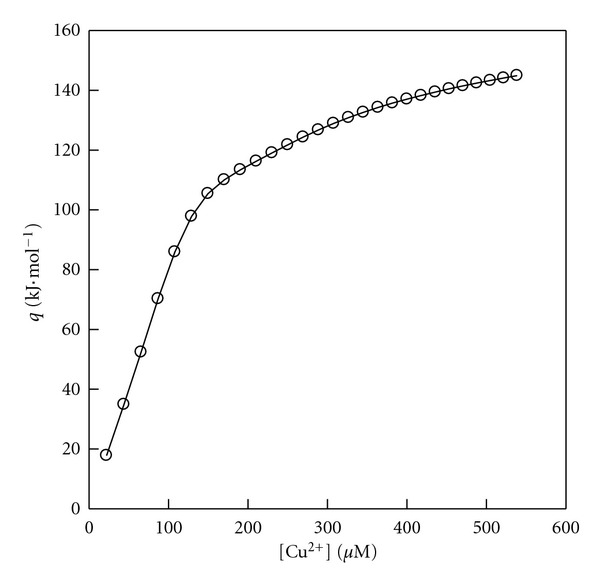
Comparison between the experimental heats (O) at 310 K, for HSA + Cu^2+^ interactions and the calculated data (lines) via (1). [Cu^2+^] values are the concentrations of [Cu(NO_3_)_2_] solution in *μ*M.

 Human serum albumin (HSA; MW = 66411 gr/mol) and tris salt are obtained from Sigma Chemical Co. The isothermal titration microcalorimetric experiments were performed with the four-channel commercial microcalorimetric system. Copper solution (4 mM) was injected by use of a Hamilton syringe into the calorimetric titration vessel, which contained 1.8 mL HSA (27.43 *μ*M). Injection of copper solution into the perfusion vessel was repeated 28 times, with 10 *μ*L per injection. The calorimetric signal was measured by a digital voltmeter that was part of a computerized recording system. The heat of each injection was calculated by the “Thermometric Digitam 3” software program. The heat of dilution of the copper solution was measured as described above except HSA was excluded. The heats of HSA + Cu^2+^ interactions have been calculated in kJmol^−1^ and shown graphically in Figure 1. 

Isothermal titration calorimetry (ITC) measurements were made on a VP-ITC ultrasensitive microcalorimeter (MicroCal, Northampton, MA) for *Aβ*(1–16) + Cu^2+^ interaction. The procedure was carried out as follows. 

**Figure 2:**
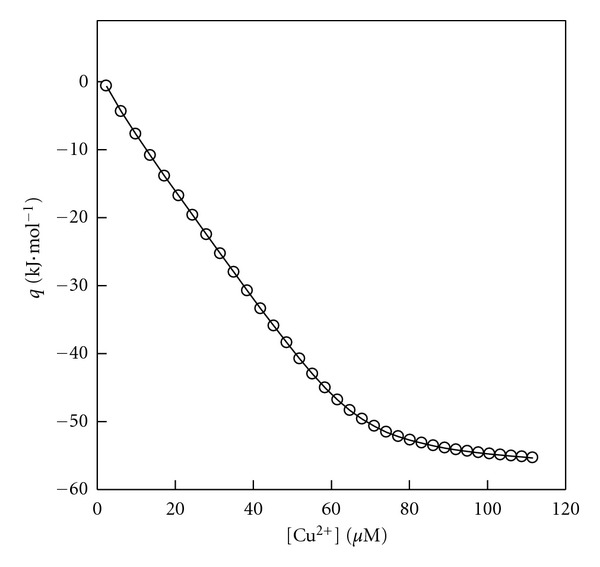
Comparison between the experimental heats (O) at 310 K, for *Aβ*(1–16) + Cu^2+^ interactions and the calculated data (lines) via (1). [Cu^2+^] values are the concentrations of [Cu(NO_3_)_2_] solution in *μ*M.

Cu^2+^ solution (0.7 mM) and *Aβ*(1–16) (75 *μ*M) solution were prepared in HEPES buffer (20 mM, 150 mM NaCl, pH 7.2, at 310 K). During the titration, 8 *μ*L of the Cu^2+^ solution was injected with 5 min intervals into the calorimetric titration vessel, which contained 1.46 mL *Aβ*(1–16). The cell was stirred at 307 rpm. The titration was conducted at 310 K. The peptide concentrations were estimated by the BCA assay (Pierce Biotechnology). Injection of Cu^2+^ solution into the vessel was repeated 30 times, with 8 *μ*L per injection. The calorimetric signal was measured by a digital voltmeter that was part of a computerized recording system. The heat of each injection was calculated by the “Thermometric Digitam 3” software program. The heats of dilution of the Cu^2+^ solution were measured as described above excluding *Aβ*(1–16). The heats of dilution of the Cu^2+^ solutions were subtracted from the enthalpies of *Aβ*(1–16) + Cu^2+^ interactions. The heats of dilution of *Aβ*(1–16) are negligible. The microcalorimeter was frequently calibrated electrically during the course of the study. The heats of *Aβ*(1–16) + Cu^2+^ interactions have been calculated in kJmol^−1^ and shown graphically in Figure 2.

## 3. Results and Discussion

We have shown previously that the heats of the ligand + HSA interactions in the aqueous solvent systems, can be calculated via the following equation [10–19]:

(1) q=qmax⁡xB′−δAθ(xA′LA+xB′LB)−(δBθ−δAθ)(xA′LA+xB′LB)xB′,

  
*q* is the heats of HSA + Cu^2+^ interactions and *q*_max⁡_ represents the heat value upon saturation of all HSA or *Aβ*(1–16). The parameters *δ*_*A*_^*θ*^ and *δ*_*B*_^*θ*^ are the indexes of the biomolecules stability in the low and high Cu^2+^ ion concentrations, respectively. *x*_*B*_′ can be expressed as follows:

(2) xB′=pxBxA+pxB,

*x*_*B*_′ is the fraction of the bound Cu^2+^ ion to the binding sites, and *x*_*A*_′ = 1 − *x*_*B*_′ is the fraction of unbound Cu^2+^ ion. We can express *x*_*B*_ fractions, as the total Cu^2+^ concentrations divided by the maximum concentration of the Cu^2+^ upon saturation of all HSA as follows:

(3) xB=[Cu2+][Cu2+]max⁡,    xA=1−xB,

[Cu^2+^] is the concentration of Cu^2+^ ions and [Cu^2+^]_max⁡_ is the maximum concentration of the Cu^2+^ ions upon saturation of all HSA. *p* > 1 or *p* < 1 indicates positive or negative cooperativity of a macromolecule for binding with a ligand, respectively; *p* = 1 indicates that the binding is noncooperative. *L*_*A*_ and *L*_*B*_ are the relative contributions of unbound and bound metal ions in the heats of dilution in the absence of HSA and can be calculated from the heats of dilution of Cu^2+^ ions in buffer, *q*_dilut_, as follows:

(4) LA=qdilut+xB(∂qdilut∂xB),  LB=qdilut+xA(∂qdilut∂xB).

The heats of HSA + Cu^2+^ and *Aβ*(1–16) + Cu^2+^ interactions, *q*, were fitted to (1) across the entire Cu^2+^ composition. In the fitting procedure, the adjustable parameter (*p*) was changed until the best agreement between the experimental and calculated data was approached (Figures 1 and 2). The binding parameters for these interactions recovered from (1) were listed in Tables 1 and 2. The agreement between the calculated and experimental results (Figure 2) is striking, and gives considerable support to the use of (1). *δ*_*A*_^*θ*^ and *δ*_*B*_^*θ*^ values for HSA + Cu^2+^ interactions are positive, indicating that in the low and high concentrations of the Cu^2+^ ions, the HSA structure is stabilized. *p* = 1 indicates that the bindingis non-cooperative. The *δ*_*A*_^*θ*^ and *δ*_*B*_^*θ*^ values for *Aβ*(1–16) + Cu^2+^ interactions are negative (unstable *Aβ*(1–16) + Cu^2+^ complex), showing that the trace amounts of copper ion can induce beta-amyloid accumulation and can significantly retard the ability to learn a difficult trace conditioning task. HSA + Cu^2+^ complex is more stable than *Aβ*(1–16) + Cu^2+^ complex, resulting in copper transfer from *Aβ*(1–16) to human serum albumin, inhibits aggregation, and reduces *Aβ*(1–16) toxicity. The negative *δ*_*A*_^*θ*^ and *δ*_*B*_^*θ*^ values indicate that *Aβ*(1–16) + Cu^2+^ complex is unstable, causing aggregation of *Aβ*(1–16) peptides, which have an influence on their fibrillization. The large and positive *δ*_*A*_^*θ*^ and *δ*_*B*_^*θ*^ values show that stable HSA + Cu^2+^ complex causes a strong tendency for transferring Cu^2+^ ions from *Aβ*(1–16) to HSA and decreasing the risk of Alzheimer's disease. These interpretations show that copper transfer from *Aβ*(1–16) + Cu^2+^ to human serum albumin inhibits aggregation and reduces *Aβ*(1–16) toxicity. Stability indexes indicate that HSA has much higher affinity for Cu^2+^ ions. Thereby, it is possible to propose that HSA is a good competitive reactant for removing Cu^2+^ ions from *Aβ*(1–16). Copper ions in solution interact strongly with human serum albumin as evidenced by large and positive *δ*_*A*_^*θ*^ and *δ*_*B*_^*θ*^ values and stabilized the HSA structure significantly. A new method is proposed to prevent amyloid plaque formation associated with Alzheimer's disease by injection of blood serum albumin in order to keep stable HSA + Cu^2+^ complex dominant against unstable *Aβ*(1–16) + Cu^2+^ ones. Our results have shown that HSA preferentially binds to toxic *Aβ*(1–16) inhibiting their growth into larger *Aβ*(1–16) assemblies.

Hopefully this suggestion proves successful, a human application would be of novel benefit for either preventing Alzheimer's disease plaque formation or possibly reverse existing plaques.

 We hypothesized that *Aβ*(1–16) aggregation induced by copper (evidenced by negative value of *δ*_*B*_^*θ*^) plays a key role in the neuropathology of Alzheimer's disease. The above interpretations are in agreement with our results. The association binding constants for copper with A*β* is much larger, while this affinity is comparatively lower for HSA. *Aβ*(1–16) structure is destabilized greatly as a result of binding to Cu^2+^ ions as evidenced by −29.967 value of *δ*_*B*_^*θ*^.

## 4. Conclusion

The large and positive *δ*_*A*_^*θ*^ and *δ*_*B*_^*θ*^ values indicate that Cu^2+^ stabilizes the HSA structure significantly. The negative *δ*_*A*_^*θ*^ and *δ*_*B*_^*θ*^ values indicate that complexes between *Aβ*(1–16) and copper ions have been proposed to be an aberrant interaction implicated in the development of Alzheimer's disease, where Cu^2+^ is involved in *Aβ*(1–16) aggregation. The precious approach is that it is possible to predict a roughly treatment using the extended solvation model in vitro.

## Figures and Tables

**Table 1:** Binding parameters for HSA+Cu^2+^ interaction. Positive  and large *δ*_*A*_^*θ*^ and *δ*_*B*_^*θ*^ values indicate that Cu^2+^ could affect the structure of HSA strongly and stabilize the HSA structure significantly.

Parameters	*T* = 310 K
*p*	1 ± 0.01
*δ*_*A*_^*θ*^	59.06 ± 0.18
*δ*_*B*_^*θ*^	58.34 ± 0.19

**Table 2:** Binding parameters for A*β*(1–16) + Cu^2+^ interaction. Negative  *δ*_*A*_^*θ*^ and *δ*_*B*_^*θ*^  values indicate that Cu^2+^ could affect the structure of A*β*(1–16) and destabilize the A*β*(1–16) structure significantly. Unstable A*β*(1–16) + Cu^2+^ complex causes the aggregation of A*β*(1–16) in the brain.

Parameters	*T* = 310 K
*p*	1 ± 0.01
*δ*_*A*_^*θ*^	−0.17 ± 0.05
*δ*_*B*_^*θ*^	−29.97 ± 0.23
